# Food products qualifying for and carrying front-of-pack symbols: a cross-sectional study examining a manufacturer led and a non-profit organization led program

**DOI:** 10.1186/1471-2458-13-846

**Published:** 2013-09-13

**Authors:** Teri E Emrich, Joanna E Cohen, Wendy Y Lou, Mary R L’Abbé

**Affiliations:** 1 Department of Nutritional Sciences, FitzGerald Building, University of Toronto, 150 College Street, Toronto, Canada; 2 Department of Health, Behavior and Society, Johns Hopkins Bloomberg School of Public Health, 2213 McElderry Street, Baltimore, USA; 3 Dalla Lana School of Public Health, University of Toronto, 155 College Street, Toronto, Canada

**Keywords:** Nutrition labelling, Front-of-pack nutrition rating systems, Nutrient criteria

## Abstract

**Background:**

Concern has been raised that the coexistence of multiple front-of-pack (FOP) nutrition rating systems in a marketplace may mislead consumers into believing that a specific food with a FOP is ‘healthier’ than foods without the symbol. Eleven summary indicator FOP systems are in use in Canada, including one non-profit developed system, the Heart and Stroke Foundation’s Health Check™, and ten manufacturer-developed systems, like Kraft’s Sensible Solutions™. This study evaluated FOP’s potential to mislead consumers by comparing the number of products qualifying to carry a given FOP symbol to the number of products that actually carry the symbol.

**Methods:**

The nutritional criteria for the Health Check™ and the Sensible Solutions™ systems were applied to a 2010–2011 Canadian national database of packaged food products. The proportion of foods qualifying for a given FOP system was compared to the proportion carrying the symbol using McNemar’s test.

**Results:**

Criteria were available to categorize 7503 and 3009 of the 10,487 foods in the database under Health Check™ and Sensible Solutions™, respectively. Overall 45% of the foods belonging to a Health Check™ category qualified for Health Check’s™ symbol, while only 7.5% of the foods carried the symbol. Up to 79.1% of the foods belonging to a Sensible Solutions™, category qualified for Sensible Solutions’s™ symbol while only 4.1% of the foods carried the symbol. The level of agreement between products qualifying for and carrying FOP systems was poor to moderate in the majority of food categories for both systems. More than 75% of the products in 24 of the 85 Health Check™ subcategories and 9 of 11 Sensible Solution™ categories/subcategories qualified for their respective symbols based on their nutritional composition.

**Conclusions:**

FOP systems as they are currently applied are not, in most instances, a useful guide to identifying healthier food products in the supermarket as many more products qualify for these systems than the number of products actually displaying these symbols on FOP, and the level of agreement between qualifying and carrying products is poor to moderate. The adoption of a single, standardized FOP system would assure consumers that all products meeting certain nutritional standards are designated by the symbol.

## Background

The World Health Organization has stated “consumers require accurate, standardized and comprehensible information on the content of food items in order to make healthy choices” [1]. To that end, mandatory nutrition labels have been adopted in more than 20 countries, including the European Union member states, Mexico, and China, and voluntary nutrition labels have been adopted in at least 11 more [2]. In Canada, regulations mandating nutrition labelling on most packaged foods were adopted 2003 in response to mounting evidence of the contribution of diet to chronic disease [3]. The Canadian Nutrition Facts table reports the amount of calories, fat, saturated and *trans* fat, cholesterol, sodium, carbohydrate, fibre, sugar, protein, vitamin A, vitamin C, calcium, and iron per serving of a food and is similar to the Nutrition Information and Nutrition Facts panels used in countries such as the United States, United Kingdom, Australia, and New Zealand. At the same time the Nutrition Facts table was adopted, Canada updated regulations for the use of nutrient content claims and established rules for the use of diet-related health claims on food products. Canada is just one of many countries, including Japan, China, Australia and New Zealand, European Union member states, and the United States, permitting some form of nutrient or health claims on food labels [4].

Not included in Canada’s 2003 regulatory revisions was another form of food label nutrition information, front-of-pack (FOP) nutrition rating systems and symbols. FOP systems provide simplified information about the nutritional characteristics of a food and have been in use internationally since American Heart Association first launched its Heart Guide initiative (1987) and Sweden’s National Food Administration created its Keyhole symbol (1989) [5]. Despite being used internationally for more than 25 years, few specific regulations are in place governing their use, although standardized FOP systems are being considered in several countries [2]. In Canada a mandatory FOP system is not presently being considered, and the only regulatory requirement currently governing the use of FOP systems is that they not be “false, misleading, or deceptive” [6]. To minimize the potential for misrepresentation, the Canadian Food Inspection Agency has issued additional guidance that FOP systems should not give the impression “that a single food or brand of food is “healthier” than … other foods not bearing the [FOP symbol]” [6].

**Figure 1:**
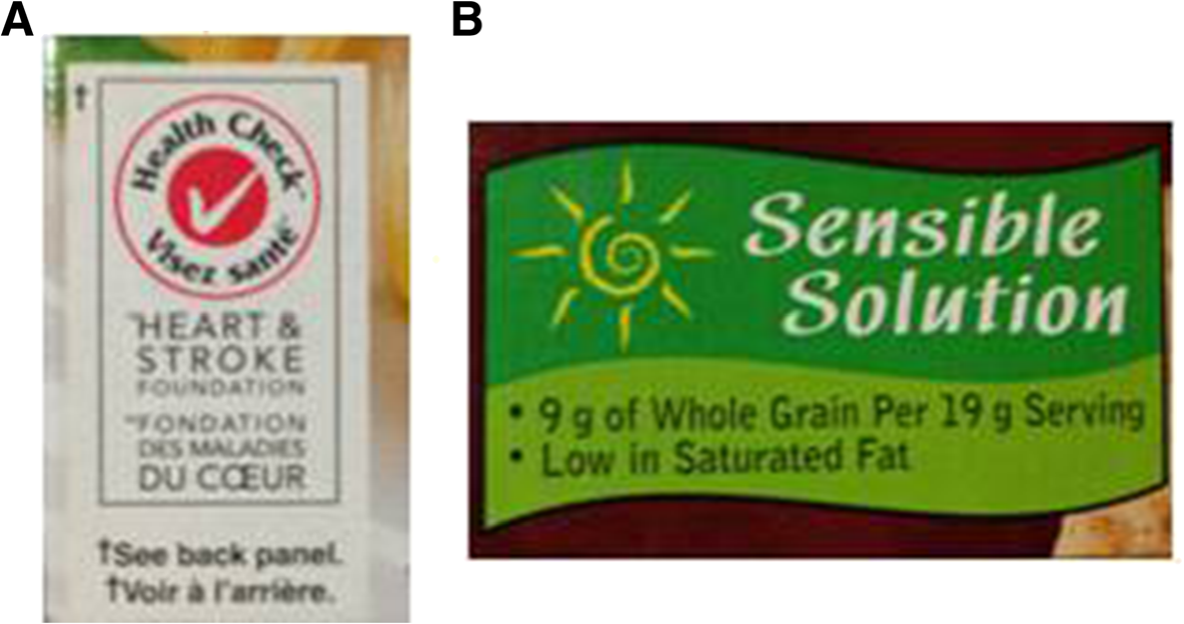
**Front-of-pack symbols evaluated in the present study. (A)** Heart and Stroke Foundation Health Check™ and **(B)** Kraft Sensible Solutions™.

Since the introduction of the Heart Guide and Keyhole programs, the number of FOP systems in the marketplace internationally has multiplied [2,5]. Each of these FOP systems has their own unique symbol and nutritional criteria to identify qualifying products. One-hundred fifty-eight unique FOP systems have been identified in the Canadian marketplace, including 11 summary indicator systems that use a single symbol on products that meet the system’s criteria [7]. Of the summary indicator systems, there was only one third-party, non-profit developed system, the Heart and Stroke Foundation of Canada’s Health Check™ symbol (Figure 1). The Health Check™ symbol can be placed on qualifying products from any manufacturer (provided the manufacturer has paid into the program) [8]. The remaining 10 systems were manufacturer or industry-developed and their symbols were placed exclusively on qualifying products of the proprietary manufacturer [7]. Examples of manufacturer-developed summary indicator systems in use in Canada include Kraft’s Sensible Solutions™ (Figure 1), Lassonde’s Health Signature^®^, Old Dutch Foods’ Snack Wise™, Pepsi’s Smart Spot™.

Many summary indicator systems are based on nutrient thresholds that establish maximum levels for nutrients to limit and minimum levels for nutrients or food components to encourage and often use different thresholds for different food categories [9,10]. The number of food categories covered by each FOP system ranges from as few as one (Snack Wise™ is only applied to snacks) to as many as 85 (Health Check™ criteria were available for 85 sub-categories found within 6 major categories at the time data were collected) [10,11]. Additional file 1 lists the Health Check™ food categories [see Additional file 1]. Sensible Solutions™ has developed criteria for the most food categories of all the manufacturer-developed summary indicator systems, with criteria available for eight major food categories and five additional subcategories [9,12]. An additional text file lists the Sensible Solutions™ food categories [see Additional file 2]. FOP systems in Canada included both absolute and relative nutrient thresholds [12,13]. Absolute thresholds establish minimum and maximum levels for nutrients and food components, whereas relative thresholds are set relative to the nutrient content of an appropriate reference product. In the latter case, products can qualify for a symbol by being lower in a nutrient like calories, fat, saturated fat, sugar, or sodium, than the reference; thus, symbols can potentially appear on foods high in saturated or *trans* fat, sugar, or sodium but low in the nutrient of interest. The nutrient criteria of summary indicator systems, and manufacturer-developed criteria in particular, whether absolute or relative have been accused of not being stringent enough [14].

As FOP systems have multiplied, so too have concerns that these systems may be confusing and misleading to consumers [5,12,13,15]. In the most comprehensive review of FOP systems to date, the Institute of Medicine concluded that the coexistence of multiple FOP systems, with different nutritional criteria, make it difficult for consumers to interpret nutrition information and to compare products [16]. Expert reports have raised further concerns about the role of FOP systems in hindering product comparisons, worrying that nutritious foods not part of a FOP program may, by default, be perceived as less healthy [12,13]. This is of concern, as many foods may not carry FOP symbols for reasons unrelated to their nutritional value. However, just how many foods are being excluded from carrying a specific FOP symbol for reasons unrelated to nutritional composition has not been examined.

This study assesses the proportion of Canadian grocery products that qualify for a Health Check™ or a Sensible Solutions™ symbol based on their nutritional composition compared to the proportion of products that actually carry these symbols in order to evaluate the potential of the current FOP systems to mislead consumers.

## Methods

The FOP systems examined in this research were the Heart and Stroke Foundation’s Health Check™ and Kraft’s Sensible Solutions™. Health Check™ was chosen as it is the only non-profit, third-party summary indicator FOP system identified to date in Canadian reports [7,12,13]. Sensible Solutions™ was chosen because it is the manufacturer-developed FOP system with nutrient criteria established for the largest number of food categories [12].

The nutrient criteria used to determine if a product qualified for the Health Check™ or Sensible Solutions™ symbol were obtained from the systems’ proprietors [9,10]. The Health Check™ criteria are based on levels of total fat, saturated and *trans* fat, sodium, carbohydrates, fibre, sugar, protein, and vitamins and minerals and the presence of fruit and vegetables and whole grains. Sensible Solutions™ criteria are based on the same nutrients and food components as Health Check™ [10], but also include criteria related to calories, cholesterol, added sugars, serving size, and functional nutritional benefits [9]. Both FOP systems use threshold criteria but differ, 1) at what level the thresholds have been set, and, 2) with respect to the application of relative thresholds. In addition to allowing products to qualify for their symbol by meeting absolute thresholds, Sensible Solutions™ also allows some products to qualify for their symbol using relative threshold criteria (provided that it passes a review by Kraft’s Nutrition Department) [9]. Examples of Health Check™ and Sensible Solution™ criteria for crackers are found in Table 1 and the remaining criteria are publicly available online from Health Check™ (http://www.healthcheck.org) and Kraft (http://www.kraftcanada.com).

**Table 1:** Nutrient criteria for crackers to qualify for Health Check™ and Sensible Solutions™ symbols

**FOP system**	**Health Check™ (11)**	**Sensible Solutions™ (12)**
Food Category	Crackers/Rusks	Cookies & Crackers
Amount of food	Per 20 g serving and per on-pack serving	Per serving
Must meet all of the following absolute threshold nutrient criteria:		
●Calories	● No criteria	● ≤ 100 calories
●Fat	● ≤ 3 g	● ≤ 30% of calories
●Saturated and trans fat	● ≤ 2 g + ≤15% of calories (combined)	● ≤ 10% of calories (combined)
	● ≤ 5% of total fat (trans)	
● Sodium	● ≤ 480 mg (per 50 g)	● ≤ 290 mg
● Added sugar	● No criteria	● ≤25% of calories
● Other	● No criteria	●A “source of” Vitamin A, C, E, calcium, magnesium, potassium, iron, protein, fibre; or,
		● Contain at least a half-serving of fruit, vegetable, or a nutritionally significant amount of whole grain; or,
		● Has a functional nutrition benefit.
Or must meet one of the following relative threshold nutrient criteria:		
● Calories	● Not applicable*	● Must be free of, or low in, one of these nutrients, or must have 25% less of one of these in comparison to the base product or an appropriate reference product
● Fat		
● Saturated fat		
		● Must be reviewed by the Nutrition Department
● Sugar		
● Sodium		

*The Health Check™ system does not use relative threshold criteria.

Data for this study were drawn from the Food Label Information Program (FLIP), a national database of food label information developed at the University of Toronto [7]. The FLIP includes the food label information from 10,487 national and private label grocery products in 23 food categories collected throughout 2010–2011 from the three largest grocery retailers in Canada (Loblaw Inc, Sobeys Inc, and Metro Inc) and one major western Canadian grocery retailer (Safeway) [10]. As previously described by Schermel et al. [7], by systematically scanning the grocery store shelves we aimed to collect every food product with a Nutrition Facts table within each of the 23 categories, including all available national and private label brands, but excluding seasonal products (e.g. egg nog) and foods from the natural health section of each store. Food products sold at multiple retailers were only purchased once and when multiple sizes of a product were available, only one size was purchased. Information recorded from the food labels into the FLIP database included the product name, nutrition information, and FOP symbols used.

Nutrition information from the Nutrition Facts table and the ingredient list were used to determine which products qualified for the FOP systems under study. Nutrients such as Vitamin E, magnesium, potassium, and folate, which are not required in the Nutrition Facts table, as well as the quantity of whole grains or servings of vegetables and fruit in the product were included in some of the criteria for both the Health Check™ and Sensible Solutions™ systems as “or” statements (i.e. products could qualify by being a source of one of these nutrients or food components or by being a source of another nutrient listed on the Nutrition Facts table) [9,10]. In this study criteria were only applied to those nutrients and food components that were available from the Nutrition Facts table or ingredient list.

All FLIP products were classified into the appropriate Health Check™ and Sensible Solutions™ food categories by a single coder and the nutrient criteria for each of these FOP systems were applied. Food categories were verified by a second coder in a random sample of 5% of products and less than 0.5% of verified products were found to be misclassified. For the Sensible Solutions™ relative nutrient criteria, the mean calorie, fat, saturated fat, sugar, and sodium content for each food category/subcategory was calculated to create the reference product used to determine if a product is lower (25%) in these nutrients. It should be noted the Kraft’s relative threshold criteria were designed to compare products to a base product (such as the original product variant) or an appropriate reference product (not publicly identified by the manufacturer). In the absence of information on the composition of the base or reference products for all products in FLIP, category reference products were established based on means for the category or subcategory.

Data were analyzed using SAS software (version 9.3, SAS Institute Inc., Cary, NC, 2011). McNemar’s test was used to compare paired proportions, specifically testing whether the proportion of products qualifying for FOP symbols was different from the proportion of products carrying FOP symbols within the same food category or subcategory. Kappa coefficient was calculated to measure the agreement between products qualifying for and carrying FOP symbols [17]. The kappa coefficient measures the difference between observed agreement and expected agreement and lies on a scale of −1 to 1, where 0.0 is considered ‘poor’ agreement, 0.2 ‘slight’, 0.4 ‘fair’ , 0.6 ‘moderate’ , 0.8 ‘substantial’ , and 1.0 ‘almost perfect’ agreement. Statistical significance level was set at p < .05, unless stated otherwise.

## Results

Criteria were available to categorize 7503 (71.5%) and 3009 (28.7%) of the 10,487 food products in FLIP under the Health Check™ and Sensible Solutions™ FOP systems, respectively. Health Check™ and Sensible Solutions™ did not have nutrient criteria established for the remaining, unclassified foods. FLIP had food products from 81 of Health Check™’s 85 subcategories [see Additional file 1]. No food products were collected from the following subcategories: Fresh fruit (unpackaged foods were not collected); Vegetarian terrines, spreads, or pates; Egg substitutes; and, Nut and/or seed bars.

**Figure 2:**
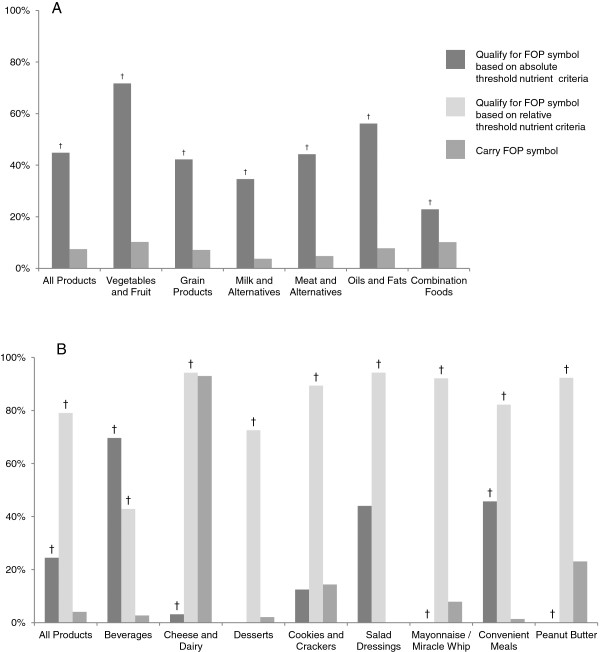
**Proportion of food products that qualified for, compared with the proportion of food products that carried, the different front-of-pack symbols. (A)** Heart and Stroke Foundation Health Check™. Criteria were available to categorize 7503 of the 10,487 food products in the FLIP under the Health Check™ system. **(B)** Kraft Sensible Solutions™. Criteria were available to categorize 3009 of the 10,487 food products in the FLIP under the Sensible Solutions™ system. † Differ significantly from the proportion of food products carrying a front-of-pack symbol p<0.05.

Details on food products qualifying for, and carrying, FOP symbols by product category are found in Figure 2. Overall, 3364 (44.8%) of the food products for which Health Check™ criteria were available met the nutrient criteria required to carry the symbol, while only 560 (7.5%) of the food products actually carried the symbol. Similarly, significantly more food products qualified for the Health Check™ symbol than carried the symbol in 56 of the 85 program subcategories. Full details of the proportion of products qualifying for, compared to carrying, the Health Check™ symbol by subcategory, including significant differences, are attached [see Additional file 1]. In most subcategories where significant differences were not observed, there was either a very low percentage of products qualifying or the database contained very few products in the subcategory. For Sensible Solutions™, when the absolute threshold nutrient criteria were used, 737 (24.5%) of products for which Sensible Solutions™ criteria were available met the eligibility criteria for the symbol; in contrast, when the relative nutrient criteria were used, 2379 (79.1%) of the same products were eligible for the symbol. Overall, only 122 (4.1%) of the products in a Sensible Solutions™ food category carried the system’s symbol. Full details by subcategory are available [see Additional file 2].

False positives (products that carried a symbol but did not meet the criteria) were rare, with more than 92% of products carrying a Health Check™ symbol meeting the relevant systems criteria. False positives were found in 13 Health Check™ subcategories with the majority of false positives in the ‘Combination foods’ subcategories. In most instances, false positives occurred because the food failed to meet the nutrient criteria per reference amount (a standard serving size established for each food category), although they met the nutrient criteria per on-pack serving. No false positives were observed with Sensible Solutions™.

There was substantial agreement (kappa >0.8) between the number of products qualifying for and carrying the Health Check™ symbol in only four subcategories, ‘dried fruit snacks’ , ‘croutons’ , ‘canned legumes’ , and ‘stuffed pasta’. Additional file 1 provides the level of agreement (kappa statistic) for all Health Check™ subcategories. Poor agreement (kappa <0.2) between qualifying and carrying products was observed in many subcategories: ‘vegetable and fruit’ (11 out of 17 subcategories); ‘grain products’ (13/19), ‘dairy products’ (7/10); ‘meat and alternative’ (14/23); ‘fats and oils’ (3/4); and, ‘combination foods’ (4/12). The highest levels of agreement between products carrying and qualifying for Sensible Solutions™ were observed when absolute threshold criteria were applied to ‘refreshment beverages’ and ‘cookies and crackers’, however even within these categories the level of agreement was poor (kappa <0.2). Additional file 2 provides the level of agreement (kappa statistic) for all Sensible Solutions™ categories and subcategories.

The Health Check™ and Sensible Solutions™ nutrient criteria were not equally discriminating in identifying “healthier” choices across food categories (Figure 2 and Additional files 1 and 2). In eight of the 18 ‘vegetable and fruit’ , seven of the 23 ‘meat and alternative’ , and two of the four ‘oils and fats’ subcategories, more than 75% of the products qualified for the Health Check™ symbol [see Additional file 1]. In contrast, in many of seven of the 12 ‘combination foods’ subcategories, less than 25% of the products qualified for the Health Check™ symbol. With respect to the Sensible Solutions™ system, fewer than 25% of the foods in each category qualified for the symbol when the absolute threshold criteria were used, with the exceptions of ‘convenient meal products’ and ‘100% juice’ [see Additional file 2]. However, when the relative threshold criteria were used, more than 70% of foods in each food category qualified for the Sensible Solutions™ symbol.

The FLIP database contained 409 products made by Sensible Solutions™, proprietor, Kraft Canada. Forty-five (11.0%) of Kraft’s products qualified for their symbol on the basis of the system’s absolute threshold criteria and 361 (88.3%) of their products qualified on the basis of the system’s relative threshold criteria. However, only 122 (29.8%) of the Kraft products in the FLIP actually carried the Sensible Solutions symbol. The lower proportion of Kraft products carrying the Sensible Solutions™, symbol relative to the number of their products that could qualify based on relative threshold criteria suggests that a significant number of products are disqualified at the required review phase by Kraft’s Nutrition Department.

## Discussion

The findings from the present study showed that significantly more products met the Health Check™ and Sensible Solutions™ nutrition rating systems’ definition of ‘healthy/healthier’ (as described in their respective nutrient criteria) than carried either of these FOP symbols in most food categories. Past research has found that, given two similar foods, one carrying the Health Check™ symbol and one without it, 80% of consumers would perceive the product with the symbol as ‘probably a better choice’ while only 4% of consumers would perceive there was ‘no real difference’ between the two products [18]. Similarly, researchers found that consumers exposed to a FOP symbol on a mousse cake perceived the cake as healthier than consumers who were given the same cake without a symbol (p = 0.004) [19]. The magnitude of perceived differences in the healthiness of foods appears to be influenced by the format of the FOP symbol [16,20-22]. Given the large number of products that qualify for, yet do not carry these symbols, our findings suggest that the two FOP systems under study may give consumers the erroneous impression that foods carrying the symbols are healthier than a similar product without these symbols – contrary to the Canadian guidance regarding the use of FOP systems [6].

When absolute threshold nutrient criteria were used, a smaller proportion of products qualified for Sensible Solutions™ than Health Check™. However, when Sensible Solutions™ relative threshold nutrient criteria were used, a larger proportion of products qualified for the Sensible Solutions™ than Health Check™. In fact, the relative threshold criteria appeared poor at differentiating between healthy and less healthy products, with a large proportion of products qualifying for the symbol in most food categories when these criteria were applied (Figure 2). Based on our findings, relative threshold nutrient criteria were less able to discriminate between products based on healthiness. However it should be noted that Kraft designed the relative nutrient criteria to be applied relative to a base product (e.g. a reduced fat Oreo cookie compared to a regular Oreo cookie) or matched with an appropriate reference product. Thus the use of category means as the reference nutrient levels for determining which products qualify based on relative thresholds is a weakness of this analysis. Furthermore, the secondary assessment by Kraft’s Nutrition Department of products that qualify based on relative threshold criteria is not documented, and could not be applied in this study.

With respect to the Health Check™ system, this study found only four subcategories where there was substantial agreement between the number of products qualifying for and carrying symbol. Considering consumers perceive products with the Health Check™ symbol as healthier than similar products without the symbol [18], our findings suggest that Health Check™ may be a useful guide to choosing healthier products for consumers in very few subcategories. Most subcategories within each of the major Health Check™ categories showed only poor agreement between products qualifying for and carrying this symbol. However, the consumer has no way to determine in which food subcategories the Health Check™ symbol identifies most products that meet the system’s definition of healthy, limiting its utility as a guide to healthier choices. However, universal implementation of a FOP system like Health Check™ or similar threshold based system to all products (not just those that have bought into the program) would allow consumers to better differentiate between healthy and less healthy food choices within all food categories. Indeed, in their 2011 report on FOP nutrition rating systems, the US Institute of Medicine recommended that an ideal FOP system should be applied to all grocery products [16].

Proponents of FOP systems suggest these systems have the potential to encourage product reformulation by manufacturers to meet their nutrient criteria [5]. The few studies that have examined this issue, including one focused on Health Check™, found that FOP systems successfully encouraged manufacturers to lower the sodium, saturated and *trans* fat, and calories in their products [23-26]. However, in 24 of 85 Health Check™ subcategories, greater than 75% of products already met the criteria, suggesting that options for reformulation would be minimal [see Additional file 1]. Similarly, when the Sensible Solutions™ relative nutrient threshold criteria were applied, more than 70% of products in many food categories qualified for the systems’ symbol. The results of this study would suggest that, within some product subcategories, the nutrient criteria of Health Check™ and Sensible Solutions™ (especially the relative thresholds) should be strengthened if they are to encourage the reformulation of more food products in a healthful way. Indeed, the Health Check™ program has been continually adjusting its nutrient criteria to encourage manufacturers to reformulate their products to reduce the amount of nutrients such as sodium and *trans* fat in the food supply [10,27,28].

The US Institute of Medicine expert committee recommended in their 2011 report that the model FOP system should be applied universally and be based on absolute thresholds for saturated and *trans* fat, sodium, and sugar for two food categories, individual foods and main dishes/meal products, to allow for the comparison of foods within and across categories [16]. In contrast to this recommendation, the two systems in this study, Health Check™ and Sensible Solutions™, had different nutrient criteria for up to 85 different subcategories, thereby limiting consumers’ ability to use these FOP systems to compare products across categories. Furthermore, the nutrient criteria used in both of these systems are based on additional nutrients beyond saturated and trans fat, sodium, and sugar, despite the Institute of Medicine’s finding that there is insufficient evidence to suggest that including such nutrients in a FOP system would be useful. Finally, the expert committee proposed that the model FOP system should take a ranked approach to nutritional guidance where, after meeting a minimum eligibility threshold, products could earn and display additional nutritional “points” based on their content of those core three nutrients. However neither system in this study offered additional ranking interpretation of the nutritional quality of products, thereby limiting their full ability to inform consumers and to promote continued product improvements.

In addition to the model FOP system proposed by the Institute of Medicine, a number of single, standardized (mandatory or voluntary) FOP systems are being proposed or adopted by governments, experts, and industry groups in countries such as the US, European Union member states, Australia and New Zealand, and South Korea [2]. The proposed systems are largely nutrient-specific, including nutrients such as saturated fat, sodium, and sugar, and display the amount per serving or per 100 g on the FOP. In addition, the use of traffic light colours to identify high (red), moderate (amber), and low (green) amounts of nutrients is under consideration in a subset of these countries. Summary indicator FOP systems, such as Health Check™ and Sensible Solutions™, are not presently being considered in any jurisdiction for universal implementation. If Canada were to consider adopting a single, standardized FOP system they may want to consider an approach more consistent with what is being proposed internationally, particularly by the Institute of Medicine. The Institute of Medicine proposes two important features that are not covered by current FOP systems; 1) they recommend a graded system with one to three stars or checkmarks awarded depending on nutritional composition; and, 2) products that don't meet the basal criteria, would carry the FOP symbol with zero stars or checkmarks.

Strengths of this study include the large number of food categories and subcategories examined, as well as the inclusion of both a non-profit led and a manufacturer led system. In addition, the FOP systems under study were quantitatively evaluated within the context of the entire food supply and evaluation was not limited to the products of the FOP system’s proprietary manufacturer or the products of manufacturers that have bought into the non-profit FOP system.

There are a few limitations to this study. First, the nutritional composition of products was based on the Nutrition Facts table and data were only available on the 14 core nutrients found in the nutrition label. Both Health Check™ and Sensible Solution™ included some criteria for nutrients and food ingredients not included in the Nutrition Facts table, thus the present study may have underestimated the number of products qualifying for these systems based on the absence of data on these nutrients and food ingredients. Furthermore, the Nutrition Facts table does not differentiate between total and added sugar, which is used in the Sensible Solutions™ system. As we were unable to differentiate between total and added sugar we applied this criteria to total sugar and, as a result, may have underestimated the total number of products that would qualify for Sensible Solutions™. In addition, reliance on nutrient content values reported in the Nutrition Facts table instead of values determined through chemical analysis may have decreased the precision of our results. However, one recent Canadian study of five food categories found no significant differences between nutrient content values reported in the Nutrition Facts table compared to values determined through chemical analysis for saturated and *trans* fat, indicating that the Nutrition Facts table values are quite precise [29].

## Conclusions

Within Canada’s current labelling environment, where FOP systems are not universally applied, nor subject to specific regulations, substantial agreement between the number of products qualifying for and carrying symbols was only found in a minority of food categories. As a result, health professionals should advise their clients that FOP symbols, in their current application, cannot be reliably used to identify food products that meet higher nutritional standards than other similar products. Overall, many more products qualified for FOP nutrition rating systems than carried them, thus supporting concerns that FOP systems could mislead consumers into thinking that products with a FOP symbol are healthier than those without when this is not actually the case.. Given the proliferation of FOP systems internationally, similar analyses should be undertaken in other countries to determine the extent to which FOP systems highlight all products that meet higher nutritional standards. As governments and industry groups implement single, voluntary FOP systems the extent of uptake should be monitored; voluntary systems may rarely be applied to products with poor nutritional quality, and as demonstrated here, if adoption is not widespread FOP systems may mislead the consumer if they believe products with symbols to be healthier than comparable products without an FOP. This analysis suggests consumers may benefit from a single, standardized FOP symbol that identifies all food products that meet a common set of nutritional standards – such as those proposed or under consideration in several countries [2].

## Abbreviations

FOP: Front-of-pack; FLIP: Food label information program.

## Competing interests

The authors declare that they have no competing interests.

## Authors’ contributions

TE participated in the design of this study, carried out all data entry, analysis, and interpretation, and drafted the manuscript. JC participated in the interpretation of the data and critically revised the manuscript. WL advised on the statistical analyses and interpretation and critically revised the manuscript. ML participated in the design of the study, advised on all aspects of the research process, and critically revised the manuscript. All authors read and approved the final manuscript.

## Authors’ information

Teri Emrich MPH, RD (PhD Candidate)

Joanna Cohen, PhD

Associate Professor

Director, Institute of Global Tobacco Control

Wendy Lou, PhD

Professor

Canada Research Chair in Statistical Methods for Health Care

Mary L’Abbé, PhD

Earle W. McHenry Professor and Chair Department of Nutritional Sciences

## Pre-publication history

The pre-publication history for this paper can be accessed here:

http://www.biomedcentral.com/1471-2458/13/846/prepub

## Supplementary Material

Additional file 1 Proportion of food products that qualified for Health Check™ compared to the proportion of food products that carried the system's symbol by subcategory (N=7503). Click here for file

Additional file 2 Proportion of food products that qualified for Sensible Solutions™ compared to the proportion of food products that carried the systems symbol by subcategory (N=3009). Click here for file
