# Supplementary material for: Food products qualifying for and carrying front-of-pack symbols: a cross-sectional study examining a manufacturer led and a non-profit organization led program
Source: BMC Public Health. 2013 Sep 13;13:846. doi: 10.1186/1471-2458-13-846 (PMC3847373; doi:10.1186/1471-2458-13-846)
Supplement: Additional file 1 — Proportion of food products that qualified for Health Check™ compared to the proportion of food products that carried the system's symbol by subcategory (N=7503). [file 1471-2458-13-846-S1.docx]

**Additional file 1 – Proportion of food products that qualified for Health Check™ compared to the proportion of food products that carried the system's symbol by subcategory (N=7503)**

| Category | N | Products carrying Health Check™  N (%) | | Products qualifying for Health Check™  N (%) | | P-Value* | Kappa Statistic (confidence interval)† |
| --- | --- | --- | --- | --- | --- | --- | --- |
| Vegetables & Fruit  Fruit Juices  Fresh Fruit  Frozen Fruit  Canned Fruit  Apple and other fruit sauces  Dried Fruit Pieces  Dried Fruit Snacks  Fresh and Frozen Vegetables (plain)  Canned Vegetables (plain)  Canned Tomatoes  Frozen and Canned Vegetables (seasoned, sauced)  Tomato Juice  Vegetable Juices and Blends  Tomato Paste  Minor Main Entrée Vegetable-Based Sauces  Vegetable-Based Dips  Frozen Fruit Bars | 554  0  6  129  33  69  38  171  162  60  27  6  35  11  93  23  7 | 47  -  0  19  6  7  14  25  7  12  1  1  6  0  1  0  0 | (8.5)  (-)  (0.0)  (14.7)  (18.2)  (10.1)  (36.8)  (14.6)  (4.3)  (20.0)  (3.7)  (16.7)  (17.1)  (0.0)  (1.1)  (0.0)  (0.0) | 317  -  6  115  18  68  14  171  160  58  6  5  18  10  49  6  0 | (57.2)  (-)  (100.0)  (89.2)  (54.6)  (98.6)  (36.8)  (100.0)  (98.8)  (96.7)  (22.2)  (83.3)  (51.4)  (90.9)  (52.7)  (26.1)  (0.0) | <.0001  -  0.0313  <.0001  0.0005  <.0001  .  <.0001  <.0001  <.0001  0.0625  0.1250  0.0005  0.0020  <.0001  0.0313  - | 0.1(0.1-0.2)  -  0.0 (0.0-0.0)  0.0 (0.0-0.1)  0.3(0.1-0.5)  0.0 (-0.0-0.0)  1.0(1.0-1.0)  0.0(0.0-0.0)  0.0(-0.0-0.0)  0.0(-0.0-0.0)  0.2(-0.2-0.6)  0.1(-0.1-0.3)  0.3(0.1-0.6)  -0.0(-0.0--0.0)  0.0(-0.0-0.1)  0.0(0.0-0.0)  - |
| Grain Products  Bread  Bread Products  Hot Breakfast Cereals  Breakfast Cereals (20-42 g per 250 mL)  Breakfast Cereals (≥43 g per 250 mL)  Very High Fibre Breakfast Cereals  Flour / Grains  Crackers / Rusks  Croutons  Rice Cakes  Waffles / Pancakes  Rice / Grains (plain)  Instant Rice (plain)  Pasta  Side Dishes – Rice, grains or potatoes (seasoned, sauced)  Side Dishes – Pasta or noodles (seasoned, sauced)  Grain-based Bars  Muffins / Muffin-Style Bars  Plain Popcorn | 185  228  57  79  145  7  6  261  53  62  73  78  5  383  132  104  178  57  53 | 20  17  4  7  7  2  0  28  5  2  2  8  2  32  7  0  7  0  3 | (10.8)  (7.5)  (7.0)  (8.9)  (4.8)  (28.6)  (0.0)  (10.7)  (9.4)  (3.2)  (2.7)  (10.3)  (40.0)  (8.4)  (5.3)  (0.0)  (3.9)  (0.0)  (5.7) | 112  91  40  29  53  7  3  115  6  18  9  78  5  242  31  1  52  11  3 | (60.5)  (39.9)  (70.2)  (36.7)  (36.6)  (100.0)  (50.0)  (44.1)  (11.3)  (29.0)  (12.3)  (100.0)  (100.0)  (63.2)  (23.5)  (1.0)  (29.2)  (15.8)  (5.7) | <.0001  <.0001  <.0001  <.0001  <.0001  0.0625  0.2500  <.0001  1.0000  <.0001  0.0156  <.0001  0.2500  <.0001  <.0001  1.0000  <.0001  0.0010  1.0000 | 0.1(0.1-0.2)  0.2(0.1-0.3)  0.0(-0.1-0.1)  0.3(0.1-0.5)  0.1(0.0-0.2)  0.0(0.0-0.0)  0.0(0.0-0.0)  0.2(0.1-0.3)  0.9(0.7-1.0)  0.2(-0.0-0.3)  0.3(-0.0-0.7)  0.0(0.0-0.0)  0.0(-0.0--0.0)  0.1(0.1-0.1)  0.3(0.1-0.4)  -0.0(-0.0--0.0)  0.2(0.1-0.3)  0.0(0.0-0.0)  0.3(-0.2-0.8) |
| Milk & Alternatives  Milk and Milk Based Drinks  Yogurts  Yogurt Based Drinks  Dairy-based dips  Fresh Cheese (plain and flavoured)  Cheese  Soy-based Cheese  Ricotta Cheese (plain)  Cottage Cheese (plain and flavoured)  Plant-based Beverages | 82  95  17  48  63  390  5  10  23  76 | 1  12  0  0  0  9  0  0  0  8 | (1.2)  (12.6)  (0.0)  (0.0)  (0.0)  (2.3)  (0.0)  (0.0)  (0.0)  (10.5) | 57  49  11  37  0  46  0  1  18  61 | (69.5)  (51.6)  (64.7)  (77.1)  (0.0)  (11.8)  (0.0)  (10.0)  (78.3)  (80.3) | <.0001  <.0001  0.0010  <.0001  -  <.0001  -  1.0000  <.0001  <.0001 | 0.0(-0.0-0.0)  0.2(0.0-0.3)  0.0(0.0-0.0)  0.0(0.0-0.0)  -  0.3(0.1-0.5)  -  -0.0(-0.0--0.0)  0.0(0.0-0.0)  0.1(0.0-0.1) |
| Meat & Alternatives  Meats / Poultry (plain, seasoned, coated)  Meats / Poultry (with sauce)  Ground Meats (plain, seasoned)  Patties, meatballs, etc.  Sausages  Deli Meats / Ham  Fish and Seafood (plain)  Fish and Seafood (seasoned or coated)  Fish and Seafood (sauced)  Canned Fish and Seafood (packed in broth or water)  Canned Fish and Seafood (seasoned, sauced)  Processed Fish  Dried Legumes  Frozen and Canned Legumes (plain)  Canned Legumes (prepared)  Tofu (plain)  Vegetarian Meat Alternatives  Vegetarian Terrines, Spreads or Pâtés  Eggs  Egg Substitutes  Nuts, Seeds or Ready to Eat Dried Legumes  Nuts and Seeds Butters  Legume-based dips | 45  103  6  126  117  213  60  104  32  86  45  7  78  95  28  15  28  0  37  0  113  39  33 | 0  4  1  6  1  4  0  1  3  1  3  2  1  2  10  3  7  -  17  -  0  1  0 | (0.0)  (3.9)  (16.7)  (4.8)  (0.9)  (1.9)  (0.0)  (1.0)  (9.4)  (1.2)  (6.7)  (28.6)  (1.3)  (2.1)  (35.7)  (20.0)  (25.0)  (-)  (46.0)  (-)  (0.0)  (2.6)  (0.0) | 13  18  6  25  5  55  49  21  7  82  26  3  78  37  10  14  17  -  37  -  68  30  23 | (28.9)  (17.5)  (100.0)  (19.8)  (4.3)  (25.8)  (81.7)  (18.3)  (21.9)  (95.4)  (57.8)  (42.9)  (100.0)  (39.0)  (35.7)  (93.3)  (60.7)  (-)  (100.0)  (-)  (60.2)  (76.9)  (69.7) | 0.0002  0.0001  0.0625  <.0001  0.1250  <.0001  <.0001  <.0001  0.1250  <.0001  <.0001  1.0000  <.0001  <.0001  1.0000  0.0009  0.0010  -  <.0001  -  <.0001  <.0001  <.0001 | -0.0(-0.0--0.0)  0.3(0.1-0.6)  0.0(0.0-0.0)  0.3(0.1-0.5)  0.3(-0.2-0.8)  0.1(0.0-0.2)  0.0(0.0-0.0)  0.1(-0.0-0.2)  0.5(0.2-0.9)  0.0(-0.0-0.0)  0.1(-0.0-0.2)  0.7(0.2-1.0)  0.0(0.0-0.0)  0.1(-0.0-0.2)  0.8(0.6-1.0)  0.0(-0.0-0.1)  0.4(0.1-0.6)  -  -0.0(-0.0--0.0)  -  -0.0(-0.0--0.0)  0.0(-0.0-0.0)  -0.0(-0.0--0.0) |
| Oils & Fats  Oils  Margarines  Light Margarines  Salad dressings | 105  47  5  267 | 4  10  2  17 | (3.8)  (21.3)  (40.0)  (6.4) | 67  39  4  128 | (63.8)  (82.9)  (80.0)  (47.9) | <.0001  <.0001  0.5000  <.0001 | 0.0(0.0-0.0)  0.1(0.0-0.2)  0.3(-0.3-0.8)  0.1(0.0-0.2) |
| Combination Foods  Soups  Dinners and Entrees / Mixed Dishes  Pizza  Vegetarian or Meat Pies  Tofu or Meat or Fish with vegetables  Stuffed Pasta  Stuffed Meat  Major Main Entrée Sauce  Potato and Pasta Salads  Other Salads  Dried Fruit and Nut Mixture  Nut and/or Seed Bars (with or without dried fruit) | 337  427  119  39  65  26  37  143  17  43  37  0 | 74  26  2  0  6  2  2  16  0  3  0  - | (22.0)  (6.1)  (1.7)  (0.0)  (9.2)  (7.7)  (5.4)  (11.2)  (0.0)  (7.0)  (0.0)  (-) | 94  81  4  0  1  3  4  62  1  17  28  - | (27.9)  (19.0)  (3.4)  (0.0)  (1.5)  (11.5)  (10.8)  (43.4)  (5.9)  (39.5)  (75.7)  (-) | 0.0005  <.0001  0.5000  -  0.1250  1.0000  0.6250  <.0001  1.0000  0.0001  <.0001  - | 0.7(0.7-0.8)  0.2(0.1-0.3)  0.7(.2-1.0)  -  -0.0(-0.0-0.0)  0.8(0.3-1.0)  0.3(-0.2-0.8)  0.3(0.2-0.4)  0.0(0.0-0.0)  0.2(-0.0-0.4)  0.0(-0.0--0.0)  - |

*P-Value for exact McNemar’s test to compare paired proportions; testing whether the proportion of products qualifying for the FOP symbol is statistically different from the proportion carrying the FOP.

† The kappa coefficient measures the difference between observed agreement and expected agreement and lies on a scale of -1 to 1, where 0.0 is considered ‘poor’ agreement, 0.2 ‘slight’, 0.4 ‘fair’, 0.6 ‘moderate’, 0.8 ‘substantial’, and 1.0 ‘almost perfect’ agreement.

“-” Indicates that there were no products available for a meaningful calculation.
