# Supplementary material for: Food products qualifying for and carrying front-of-pack symbols: a cross-sectional study examining a manufacturer led and a non-profit organization led program
Source: BMC Public Health. 2013 Sep 13;13:846. doi: 10.1186/1471-2458-13-846 (PMC3847373; doi:10.1186/1471-2458-13-846)
Supplement: Additional file 2 — Proportion of food products that qualified for Sensible Solutions™ compared to the proportion of food products that carried the systems symbol by subcategory (N=3009). [file 1471-2458-13-846-S2.docx]

**Additional file 2 – Proportion of food products that qualified for Sensible Solutions™ compared to the proportion of food products that carried the systems symbol by subcategory (N=3009)**

| Category | N | Products Carrying Sensible Solutions**™**  N (%) | | Absolute threshold criteria | | | | Based on Relative threshold criteria | | | |
| --- | --- | --- | --- | --- | --- | --- | --- | --- | --- | --- | --- |
|  |  |  |  | Products qualifying for Sensible Solutions**™**  N (%) | | P-Value* | Kappa coefficient  (confidene interval) † | Products qualifying for Sensible Solutions**™**  N (%) | | P-Value* | Kappa coefficient† |
| Beverages  100% Juice‡  Refreshment Beverages | 317  238 | 0  15 | (0.0)  (6.3) | 216  4 | (68.1)  (1.7) | <.0001  0.0074 | 0.0 (0.0-0.0)  0.2 (-0.1-0.4) | -  238 | (-)  (100.0) | -  <.0001 | -  0.0 (0.0-0.0) |
| Cheese and Dairy  Natural and processed cheese  Cream cheese  Grated parmesan, Romano cheese, Cheese spreads and dip§ | 413  57  69 | 5  0  0 | (1.2)  (0.0)  (0.0) | 17  0  - | (4.1)  (0.0)  (-) | 0.0169  -  - | -0.0 (-0.0--0.0)  -  - | 404  40  64 | (97.8)  (70.1)  (92.8) | <.0001  <.0001  <.0001 | 0.0 (-0.0-0.0)  0.0 (0.0-0.0)  -0.0 (-0.0--0.0) |
| Desserts | 142 | 3 | (2.1) | 0 | (0.0) | 0.2500 | -0.0 (-0.0--0.0) | 103 | (72.5) | <.0001 | 0.0 (-0.0-0.0) |
| Cookies and Crackers | 536 | 77 | (14.4) | 67 | (12.5) | 0.3682 | 0.2 (0.1-0.3) | 479 | (89.4) | <.0001 | 0.0 (0.0-0.0) |
| Salad Dressings | 228 | 0 | (0.0) | 1 | (0.44) | 1.0000 | 0.0 (0.0-0.0) | 215 | (94.3) | <.0001 | 0.0 (0.0-0.0) |
| Mayonnaise and Miracle Whip | 38 | 3 | (7.9) | 0 | (0.0) | 0.2500 | 0.0 (0.0-0.0) | 35 | (92.1) | <.0001 | 0.0 (-0.0-0.0) |
| Convenient Meals | 945 | 13 | (1.4) | 432 | (45.7) | <.0001 | 0.0 (0.0-0.0) | 777 | (82.2) | <.0001 | 0.0 (0.0-0.0) |
| Peanut Butter | 26 | 6 | (23.1) | 0 | (0.0) | 0.0313 | 0.0 (0.0-0.0) | 24 | (92.3) | <.0001 | 0.0 (-0.0-0.1) |

*P-Value for exact McNemar’s test to compare paired proportions; testing whether the proportion of products qualifying for the FOP symbol is statistically different from the proportion carrying the FOP.

†The kappa coefficient measures the difference between observed agreement and expected agreement and lies on a scale of -1 to 1, where 0.0 is considered ‘poor’ agreement, 0.2 ‘slight’, 0.4 ‘fair’, 0.6 ‘moderate’, 0.8 ‘substantial’, and 1.0 ‘almost perfect’ agreement.

‡Sensible Solutions™ does not have relative threshold criteria established for 100% Juice.

§ Sensible Solutions™ does not have absolute threshold criteria established for Grated parmesan, Romano cheese, Cheese spreads and dip.

“-” Indicates that there were no products available for a meaningful calculation.
